# ﻿*Sinojohnstoniahenanensis* (Boraginaceae), a new species from Henan, China

**DOI:** 10.3897/phytokeys.261.161917

**Published:** 2025-08-05

**Authors:** Li-Qiong Jiang, Yuan Luo, Jian-Ping Gong, Nai-Yu Zhang, Pan Li, Wen-Bo Liao, Lu-Xian Liu

**Affiliations:** 1 College of Life Sciences, The Observation and Research Field Station of Taihang Mountain Forest Ecosystems of Henan Province, Henan Normal University, Xinxiang, 453007, Henan, China Henan Normal University Xinxiang China; 2 Forestry and Grassland Bureau of Lancang Lahu Autonomous County, Pu’er, 665600, Yunnan, China Forestry and Grassland Bureau of Lancang Lahu Autonomous County Yunnan China; 3 Laboratory of Systematic and Evolutionary Botany and Biodiversity, College of Life Sciences, Zhejiang University, Hangzhou, 310058, Zhejiang, China Zhejiang University Hangzhou China; 4 State Key Laboratory of Biocontrol and Guangdong Provincial Key Laboratory of Plant Stress Biology, School of Life Sciences, Sun Yat-sen University, Guangzhou, 510275, Guangdong, China Sun Yat-sen University Guangzhou China

**Keywords:** Boraginaceae, Henan, morphology, new species, *Sinojohnstonia*

## Abstract

*Sinojohnstoniahenanensis*, a new species from the Muzhaling Scenic Area in Henan Province, China, is described and illustrated. It is morphologically similar to *S.chekiangensis* and *S.ruhuaii* but differs from the former in the size of the basal leaves, calyx size, color and size of the corolla, and length of the style; and from the latter in cauline petiole length, calyx size, color and size of the corolla, stamen insertion, filament length, and style length. Additionally, the conservation status and other pertinent notes are provided.

## ﻿Introduction

*Sinojohnstonia* Hu (Boraginaceae) is a small genus endemic to China, currently comprising four species: *S.plantaginea* Hu, *S.chekiangensis* (Migo) W.T. Wang ex Z.Ying Zhang, *S.moupinensis* (Franch.) W.T. Wang ex Z.Ying Zhang, and *S.ruhuaii* W.B. Liao & Lei Wang. *Sinojohnstonia* species are primarily distributed across northwestern to central China, extending eastward and southwestward (Wang and Liao 2014). They occur in shaded, moist habitats such as ravines, forests, and rocky slopes, at elevations ranging from 400 to 2700 m (Zhu et al. 1995; Wang and Liao 2014). The genus was first described by Hu (1936) based on specimens collected from Sichuan Province, southwest China. In the *Flora Reipublicae Popularis Sinicae*, the Boraginaceae was divided into four subfamilies: Boraginoideae, Cordioideae, Ehretioideae, and Heliotropioideae (Wang 1989). *Sinojohnstonia* was classified within the tribe Trigonotideae of the subfamily Boraginoideae, based on characteristics such as its 4-lobed ovary, terminal style, and gynobase morphology (Wang 1989). Subsequently, Chacón et al. (2016) divided the Boraginaceae into three subfamilies—Echiochiloideae, Boraginoideae, and Cynoglossoideae—based on phylogenetic inference from four chloroplast DNA regions (*trnL*, *trnL-F*, *rps16*, and *trnS-G*) and nuclear ribosomal internal transcribed spacer (nrITS) sequences. Further analysis based on nrITS revealed that *Sinojohnstonia* should be classified into subtribe Bothriosperminae (Cynoglosseae, Cynoglossoideae). Morphologically, *Sinojohnstonia* can be distinguished from other genera of Boraginaceae by its ovate-cordate leaves, an extremely enlarged fruiting calyx enclosing the fruit, and tetrahedral nutlets with cupular emergences (Zhu et al. 1995).

On 29 April 2025, Mr. Hui Liu, a plant enthusiast, shared photographs of a plant taken at the Muzhaling Scenic Area in Song County, Henan Province, to the WeChat group named “Henan Plants Chat Group” and inquired about its species identification. Based on morphological characteristics—specifically, an ovate-cordate leaf blade, an extremely enlarged fruiting calyx enclosing the fruit, and nutlets with an adaxial membranous cupular emergence—we classify this taxon within *Sinojohnstonia* (Boraginaceae) and suggest it constitutes a new species. Subsequent field investigations on 2 May 2025 revealed that this taxon prefers shaded, moist montane forests and exhibits flowers morphologically distinct from all congeneric species. Following detailed morphological analysis, we confirm that it constitutes a new species of *Sinojohnstonia*.

## ﻿Methods

Field observations and collections of the new species were conducted at Muzhaling Scenic Area (Henan Province, China) on 2 May 2025. Protologues of all published names and related taxonomic and floristic literature on *Sinojohnstonia* were reviewed (Hu 1936; Zhang ZY 1983; Wang 1989; Zhu et al. 1995; Wang and Liao 2014).

Specimens from 15 herbaria (BNU, CDBI, CSFI, HIB, IBSC, JJF, JIU, KUN, LBG, NAS, PE, SM, SYS, WCSBG, and WUK; abbreviations follow Thiers 2025) and our field collections were examined. Additionally, images of specimens (including type specimens) and living plants of *Sinojohnstonia* from the Chinese Field Herbarium (CFH, https://www.cfh.ac.cn/), Global Biodiversity Information Facility (GBIF, https://www.gbif.org/), Chinese Virtual Herbarium (CVH, https://www.cvh.ac.cn/), and Plant Photo Bank of China (PPBC, http://ppbc.iplant.cn/) were checked. Morphological comparisons between the new species and other *Sinojohnstonia* taxa (*S.chekiangensis*, *S.ruhuaii*, *S.plantaginea*, and *S.moupinensis*) were performed, leveraging our prior field observations and specimen examinations. Flowers and nutlets were photographed using a ZOOM645S stereomicroscope (Jiangnan Yucheng Optical Instrument Co., Ltd., China). Morphological descriptions of the new species follow the terminology used by Zhu et al. (1995) and Wang and Liao (2014).

## ﻿Result

### ﻿Taxonomic treatment

#### ﻿ Sinojohnstonia henanensis

Taxon classificationPlantaeBoraginalesBoraginaceae

L.X.Liu & P.Li sp. nov.

AF31DDC4-7DA2-530D-A2AF-AAA5AAEF0572

urn:lsid:ipni.org:names:77366495-1

Figs 1 , 2 , 3 , 4

##### Type.

Henan Province, Luoyang City, Song County, Checun Town, Muzhaling Scenic Area, in forests, shaded moist loose soil areas, ca. 1870 m, 33°44'33.48"N, 112°14'28.13"E, 3 May 2025, *Liu LX & Yu J LLX20250503* (holotype: ZM; isotype: HZU).

##### Diagnosis.

*Sinojohnstoniahenanensis* is morphologically similar to *S.chekiangensis* and *S.ruhuaii* but differs from the former in its basal leaves with petioles 1–11 cm, lamina 0.7–6 × 0.6–4 cm (vs. with petioles 10–18 cm, lamina 3–12 × 1.5–9 cm), calyx in flower ca. 2.5–3.5 × 0.5–1.2 mm, obviously shorter than corolla tube (vs. in flower ca. 3–7 × 1.4–2.7 mm, slightly longer than corolla tube), corolla blue-purple, slightly pinkish white, ca. 6–10 mm, lobes ca. 1.8–2.5 mm (vs. white or light reddish, ca. 10 mm, lobes ca. 3–4 mm), and style ca. 6–8 mm (vs. ca. 3–6 mm), and from the latter in its calyx in flower obviously shorter than corolla tube (vs. in flower slightly longer than corolla tube), corolla blue-purple, slightly pinkish white, ca. 6–10 mm (vs. white or light reddish white, ca. 5–6 mm), anthers exserted from the corolla tube, upon the throat appendages, filaments ca. 2–4 mm (vs. anthers exserted somewhat from the corolla tube, but below the throat appendages, filaments ca. 0.5 mm), and style ca. 6–8 mm (vs. ca. 2.5–4 mm). Their differences are summarized in Table 1.

**Table 1.** Morphological features distinguishing *Sinojohnstoniahenanensis*, *S.chekiangensis*, *and S.ruhuaii*.

Characters	*Sinojohnstoniahenanensis*	*S.chekiangensis*	*S.ruhuaii*
Basal leaves size	with petioles 1–11 cm, lamina 0.7–6 × 0.6–4 cm	with petioles 10–18 cm, lamina 3–12 × 1.5–9 cm	with petioles 5–10 cm, lamina 4–6 × 2–3 cm
Cauline leaves size	with petioles 0.6–3 cm, lamina 1–4.5 × 0.8–3.5 cm	with petioles 0.3–4 cm, lamina 1–6 × 0.3–4 cm	with petioles 1–8 cm, lamina 2–6 × 1–3 cm
Calyx	calyx in flower ca. 2.5–3.5 × 0.5–1.2 mm, obviously shorter than corolla tube, in fruit enlarging to 6–8 × 1.7–3 mm	calyx in flower ca. 3–7 × 1.4–2.7 mm, slightly longer than corolla tube, in fruit enlarging to 6–10 × 2–4 mm	calyx in flower ca. 3–3.5 mm, slightly longer than corolla tube, in fruit enlarging to 8–10 mm
Corolla	blue-purple, slightly pinkish white, ca. 6–10 mm	white or light reddish, ca. 10 mm	white or light reddish white, ca. 5–6 mm
Corolla limb	distinctly shorter than corolla tube, lobes ca. 1.8–2.5 mm	distinctly shorter than corolla tube, lobes ca. 3–4 mm	slightly shorter than or nearly as long as corolla tube, lobes ca. 3–4 mm
Stamens	anthers exserted from the corolla tube, upon the throat appendages, filaments ca. 2–4 mm, anthers ca. 0.4–0.7 mm	anthers exserted from the corolla tube, upon the throat appendages; filaments ca. 3 mm; anthers ca. 0.9 mm,	anthers exserted somewhat from the corolla tube, but below the throat appendages, filaments ca. 0.5 mm, anthers ca. 0.6–0.8 mm
Style	ca. 6–8 mm	ca. 3–6 mm	ca. 2.5–4 mm

##### Description.

Herbs with several rhizomes, stems several, prostrate to spreading, up to 20 cm tall. Leaves alternate the blade ovate-elliptical, base cordate or slightly flattened truncate, apex obtuse or acuminate, densely strigose abaxially and adaxially; basal leaves several, with petioles 1–11 cm, lamina 0.7–6 × 0.6–4 cm; cauline leaves, with petioles 0.6–3 cm, lamina 1–4.5 × 0.8–3.5 cm. Inflorescences terminal, racemose with 1 or 2 branches, ca. 1–5 cm, densely short strigose, up to 9- or 10-flowered, ebracteate; pedicel ca. 1–10 mm long. Calyx in flower ca. 2.5–3.5 × 0.5–1.2 mm, obviously shorter than corolla tube, 5-parted to base; lobes linear-lanceolate; densely strigose abaxially and adaxially, in fruit enlarging to 6–8 × 1.7–3 mm and becoming saccate. Corolla blue-purple, slightly pinkish white, campanulate, ca. 6–10 mm, glabrous; corolla limb 5-parted, spreading, distinctly shorter than corolla tube; lobes ovate, ca. 1.8–2.5 mm long; throat appendages 5, semi-obicular, ca. 0.2 mm; stamens 5, inserted on upper part of corolla tube, anthers exserted from the corolla tube, upon the throat appendages; filaments ca. 2–4 mm, anthers oblong, ca. 0.4–0.7 mm. Ovary deeply 4-lobed; style ca. 6–8 mm; stigma capitate. Fruit a nutlet, tetrahedral, 3–3.5 × 1–2 mm, sparsely pubescent; nutlet with abaxial membranous margin-inflexed cupular emergence, attachment scar slightly below middle of adaxial surface; seeds ca. 2 × 1 mm.

##### Phenology.

Flowering and fruiting period from April to May.

##### Etymology.

The specific epithet is derived from its distribution in Henan Province, China.

##### Vernacular name.

We propose a Chinese name, Hé nán chē qián zǐ cǎo (河南车前紫草).

##### Distribution and habitat.

*Sinojohnstoniahenanensis* has been found only in the Muzhaling Scenic Area of Song County, Henan Province. It grows in forests with shaded, moist, and loose soil areas at an elevation of approximately 1870 meters.

##### IUCN conservation status.

Currently, *Sinojohnstoniahenanensis* is only known from a single location in Muzhaling Scenic Area of Song County, Henan Province. During the present survey, only a few mature plants (fewer than 100) were observed. The extent of occurrence for *S.henanensis* is less than 100 km^2^. The plants are directly threatened by human disturbances such as tourism. Following the IUCN (2024) criteria B and C, a preliminary conservation assessment classifies *S.henanensis* as Critically Endangered (CR).

## ﻿Discussion

**Figure 1.**
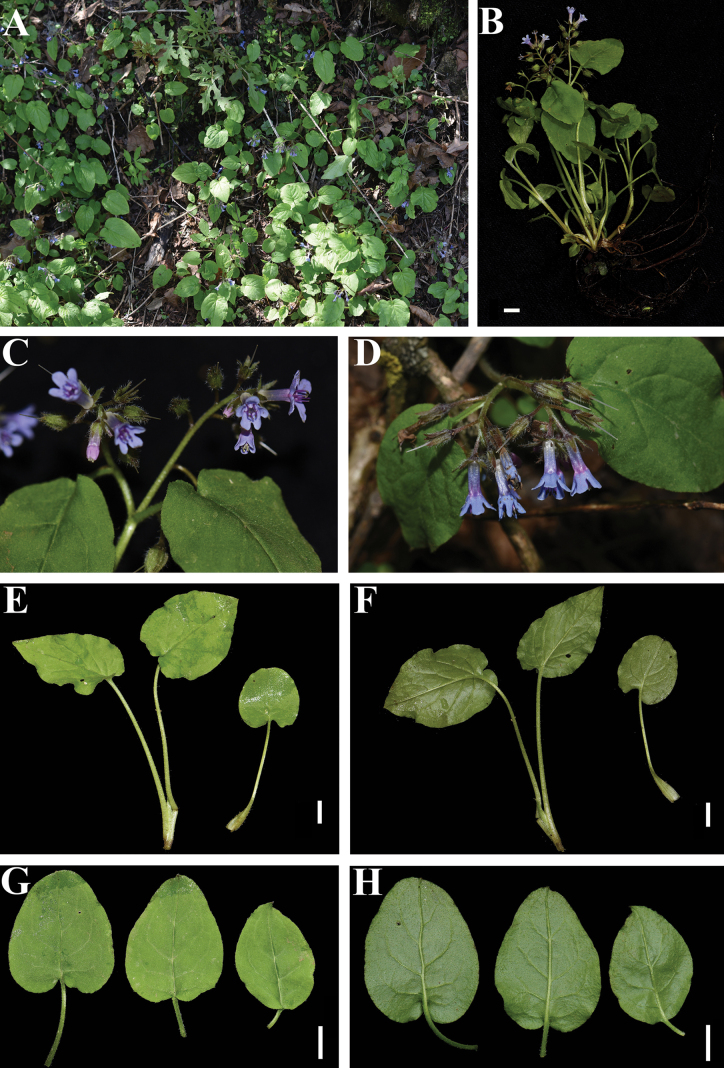
*Sinojohnstoniahenanensis* L.X. Liu & P. Li, sp. nov. A. Habitat; B. Habit; C. Front view of inflorescences; D. Side view of inflorescences; E, F. Basal leaves (E and F show adaxial and abaxial leaf surface); G, H. Cauline leaves (G and H show adaxial and abaxial leaf surface). Photographed by L.-X. Liu (A and D) and L.-Q. Jiang (B, C, and E–H). Scale bars: 1 cm.

**Figure 2.**
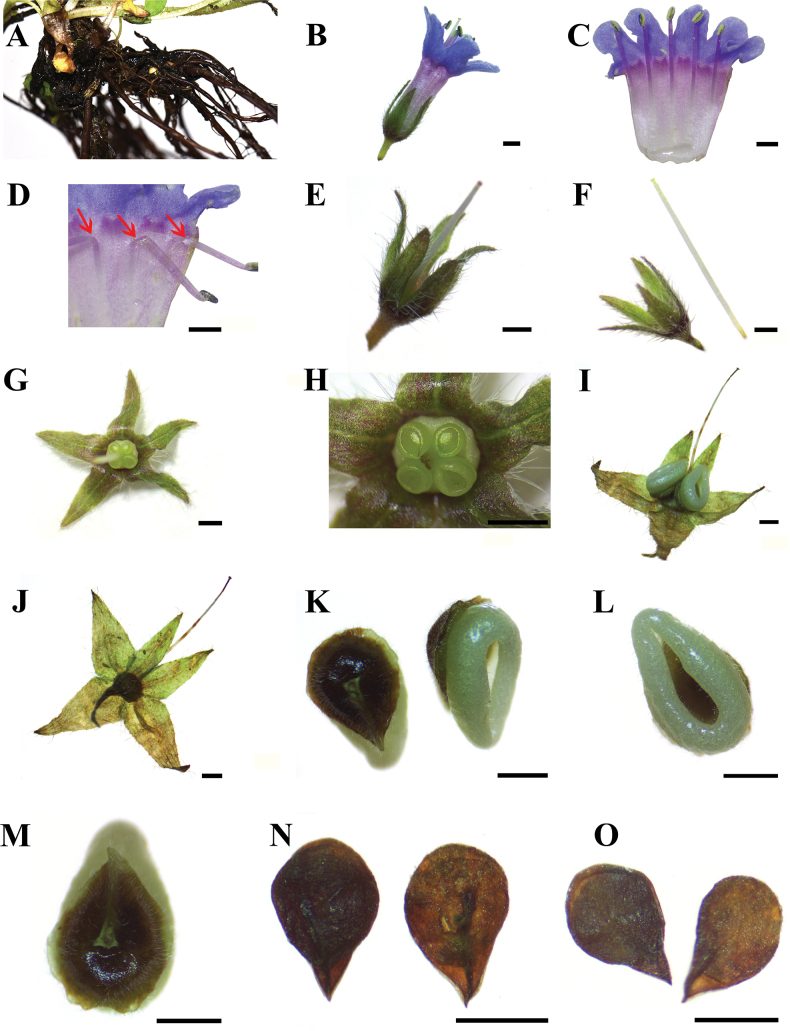
*Sinojohnstoniahenanensis* L.X. Liu & P. Li, sp. nov. A. Rhizomes; B. Calyx and corolla; C. Opened corolla; D. Position of stamens (marked with red arrow); E, F. Side view of calyx and style; G. Front view of calyx and style; H. Deeply 4-lobed ovary; I. Front view of fruits and enlarging calyx; J. Abaxial view of enlarging calyx; K–M. Adaxial and abaxial fruits; N. Adaxial seeds; O. Abaxial seeds. Photographed by L.-Q. Jiang. Scale bars: 1 mm.

**Figure 3.**
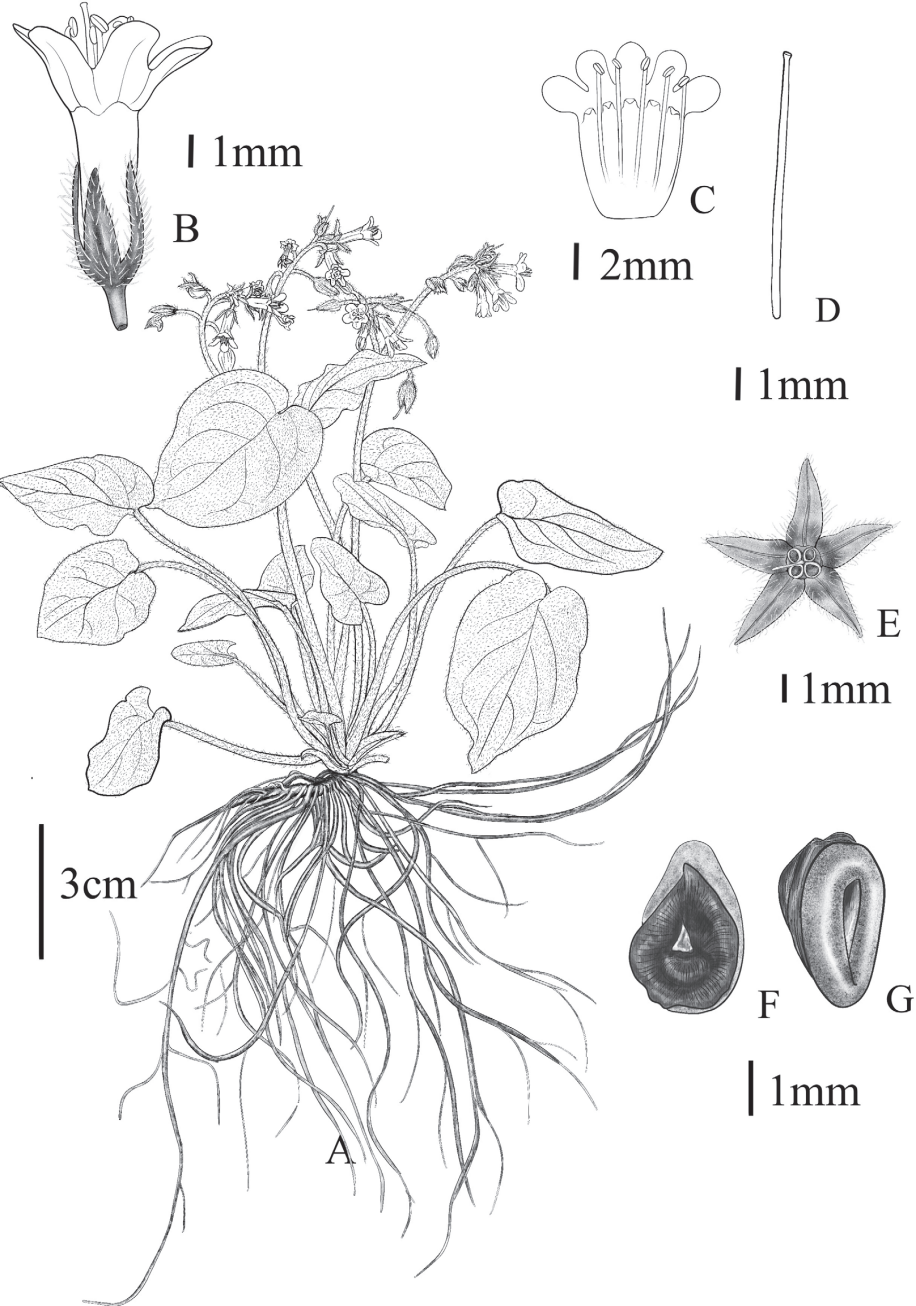
*Sinojohnstoniahenanensis* L.X. Liu & P. Li, sp. nov. A. Plant; B. Flower with calyx and corolla; C. Corolla and stamens; D. Style; E. Front view of calyx and style; F. Ventral view of nutlet; G. Dorsal view of nutlet. Drawn by Y. Luo.

**Figure 4.**
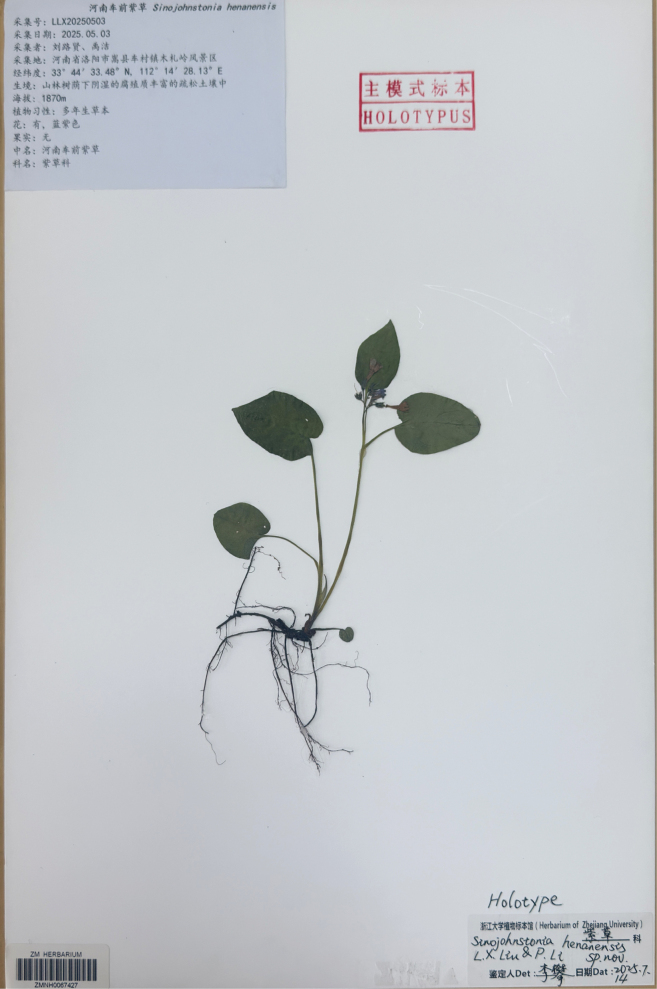
Holotype of *Sinojohnstoniahenanensis* L.X.Liu & P. Li.

*Sinojohnstonia* is a small genus consisting of four currently described species. Morphologically, *S.henanensis* is most closely related to *S.chekiangensis* and *S.ruhuaii*. Though *S.henanensis* resembles *S.chekiangensis* in leaf shape, cauline leaf size, position of the anthers, length of filaments, and nutlet size, they can readily be distinguished by the size of basal leaves, calyx size, color and size of corolla, and length of style (Table 1, Figs 1–4). The new species differs from *S.chekiangensis* mainly in its basal leaves with petioles 1–11 cm, lamina 0.7–6 × 0.6–4 cm (vs. with petioles 10–18 cm, lamina 3–12 × 1.5–9 cm in *S.chekiangensis*), calyx in flower ca. 2.5–3.5 × 0.5–1.2 mm, obviously shorter than corolla tube, in fruit enlarging to 6–8 × 1.7–3 mm (vs. in flower ca. 3–7 × 1.4–2.7 mm, slightly longer than corolla tube, in fruit enlarging to 6–10 × 2–4 mm in *S.chekiangensis*), corolla blue-purple, slightly pinkish white, ca. 6–10 mm, lobes ca. 1.8–2.5 mm (vs. white or light reddish, ca. 10 mm, lobes ca. 3–4 mm in *S.chekiangensis*), and style ca. 6–8 mm (vs. ca. 3–6 mm in *S.chekiangensis*). Both *S.henanensis* and *S.ruhuaii* have similar leaf shapes, basal leaf sizes, inflorescences, and nutlet sizes. The most noteworthy difference between the two species is in the corolla and stamens, which are blue-purple, slightly pinkish white, ca. 6–10 mm, corolla limb distinctly shorter than corolla tube, lobes ca. 1.8–2.5 mm, and anthers exserted from the corolla tube, upon the throat appendages, filaments ca. 2–4 mm in *S.henanensis*, but white or light reddish white, ca. 5–6 mm, slightly shorter than or nearly as long as corolla tube, lobes ca. 3–4 mm, and anthers exserted somewhat from the corolla tube, but below the throat appendages, filaments ca. 0.5 mm in *S.ruhuaii*. Meanwhile, cauline leaves of *S.henanensis* have 0.6–3 cm long petioles, whereas those of *S.ruhuaii* have 1–8 cm long petioles, calyx of this new species shorter than corolla tube, in fruit enlarging to 6–8 mm, style ca. 6–8 mm long; nevertheless, those of *S.ruhuaii* slightly longer than corolla tube, in fruit enlarging to 8–10 mm, style ca. 2.5–4 mm long. Detailed morphological differences between them are summarized in Table 1.

Although several distinct morphological characters differentiate *Sinojohnstoniahenanensis* from closely related species, its precise phylogenetic placement requires confirmation through robust plastome- and/or nuclear gene-based phylogenetic analyses (Liu et al. 2020, 2021; Jin et al. 2024). Recent advancements in bioinformatic methodologies have further significantly improved the resolution of complex, web-like phylogenetic relationships (Liu et al. 2022; Jin et al. 2023; Lin et al. 2025; Xie et al. 2025). These novel approaches and successful applications provide essential tools for unraveling the speciation process in *Sinojohnstoniahenanensis* in future studies.

### ﻿The diagnostic key for *Sinojohnstonia*

**Table:** 

1	Stamens included within the corolla tube; corolla limb distinctly longer than tube; throat appendages basally connected to the upper corolla tube and higher than stamens; nutlets pubescent; plants without rhizomes	**1. *S.moupinensis***
–	Stamens exserted from the corolla tube; corolla limb slightly longer to shorter than tube; throat appendages basally connected to the lower corolla lobes; nutlets glabrous or sparsely pubescent; plants with rhizomes	**2**
2	Corolla lobes narrowly triangular, ca. as long as tube; stamens inserted on throat between appendages, slightly shorter to longer than corolla	**2. *S.plantaginea***
–	Corolla lobes ovate to oblong; stamens inserted on the upper corolla tube	**3**
3	Corolla lobes slightly shorter than or nearly as long as tube; stamens exserted somewhat from corolla tube and below throat appendages	**3. *S.ruhuaii***
–	Corolla lobes distinctly shorter than tube; stamens distinctly exserted from corolla tube and upon throat appendages	**4**
4	Corolla white or light reddish, lobes ca. 3–4 mm, style ca. 3–6 mm	**4. *S.chekiangensis***
–	Corolla blue-purple, slightly pinkish white, lobes ca. 1.8–2.5 mm, style ca. 6–8 mm	**5. *S.henanensis***

### ﻿Additional specimens examined

***Sinojohnstoniachekiangensis***: China • Anhui: • Baimazhai, Wetlands along ditches, elev. 650 m, 30 April 1983, *J. Z. Shao 834027* (PE!); • Huangshan, On north-facing slopes in the forest understory along ditches, elev. 720 m, 3 April 1983, *X. S. Shen 49* (PE!); • Huangshan, in forest understory, elev. 1000 m, 9 May 1982, *D. Q. Wang 3915* (PE!); • Huangshan, On valley margins, elev. 1200 m, 6 May 1986, *J. Z. Shao 86417* (PE!); • Huangshan, 10 April 1984, *T. S. Zhou & G. H. Ye 7024* (PE!); • Jinzhai County, Tiantangzhai Scenic Area, under the trees in the valley, elev. 689 m, 31°9'48.38"N, 115°46'54.88"E, 16 April 2021, *X. X. Zhu et al. ZXX21360* (HIB!); • Jinzhai County, Tiantangzhai Scenic Area, under the trees by the stream in the valley, elev. 800–1100 m, 25 April 1999, *Q. Wang & G Yao 99047* (NAS!); • Jinzhai, Baimazhaigongshe, in shaded, humid microhabitats, elev.720 m, 3 April 1983, *X. S. Shen Shen 0049* (PE!) ; • Jinzhai County, on wet valley, elev. 1000 m, 22 April 1984, *Z. L. Chen 45* (NAS!); • Jinzhai County, Huashi Township, Baimazhai Forest Farm, on wet valley, elev. 650 m, 21 September 1981, *M. B. Deng 81102* (NAS!); • Jinzhai County, Baimazhai Forest Farm, on wet valley, elev. 800 m, 11 May 1984, *G. Yao 8870* (NAS!); • Jinzhai County, Baimazhai Forest Farm, on wet valley, elev. 1000 m, 25 May 1986, *G. Yao 9778* (NAS!); • Shitai County, Qidou Town, on the stone, elev. 356.32 m, 30°21'36.00"N, 117°54'0.00"E, 8 March 2016, *J. W. Shao et al. ANUB00344* (ANUB!); • Shitai, Qijing, along roads on hillsides, elev. 890 m, 4 April 1983, *D. Q. Wang & T. S. Zhou 5477* (PE!). • Henan: • Lingbao City, on the up-mountain path of Laoya Cha, 13 June 2017, *J. Wang 2017613025* (HEAC!); • Lingbao City, Xiaoqinling Nature Reserve, Xiangguan Trail, by the roadside and on the mountain slopes, 9 May 2015, *S. X. Zhu et al. 150509017* (AU!); • Shihe District, Jigong Mountain, subtropical coniferous and broad-leaved mixed forest, elev. 359 m, 31°48'08.20"N, 114°03'50.29"E, 12 March 2019, *X. X. Zhu et al. ZXX19049* (HIB!); • Xinyang, Jigongshan, 2 May 1984, *Botanical Resource Expedition D0071* (PE!). • Hubei: • Shennongjia, Dajiuhu National Wetland Park, by the roadside, facing the downhill slope, elev. 1471.71 m, 31°26'8.94"N, 110°10'21.94"E, 21 April 2021, *C. Zhang & L. D. Yang Zhang 711* (BNU!); • Wufeng County, Houhe National Nature Reserve Scenic Area, in the grass under the broad-leaved trees in the canyon, elev. 0 m, 30°05'09.57"N, 110°33'3.46"E, 22 April 2017, *D. G. Zhang et al. zdg, wyq, zxq 0346* (JIU!); • Yingshan County, elev. 1108 m, 31°0'34"N, 116°2'37"E, 26 April 2015, *B. J. Ge et al. GBJ04392* (CSH!). • Hunan: • Baojing County, Baiyun Mountain, in the thickets, 27 April 2009, *D. G. Zhang YD11066* (JIU!); • Baojing County, Baiyun Mountain, under the trees, 3 July 2017, *D. G. Zhang 10394* (JIU!); • Changsha city, Liuyang City, Dawei Mountain National Forest Park, on mountain slopes with humus soil, roadside, 15 September 2014, *W. B. Liao et al. LXP-13-17025* (SYS!); • Hengshan,Within dense forests along mountain streams in valleys, elev. 0–630 m, 25 May 1949, *S. Q. Chen 3277* (IBSC!); • Xiangxi Tujia and Miao Autonomous Prefecture, Longshan County, Luota Stone Forest, elev. 932 m, 29°10'38"N, 109°28'25"E, 13 May 2013, *D. K. Tian et al. LS-1777* (CSH!); • Xiangxi Tujia and Miao Autonomous Prefecture, Longshan County, elev. 932 m, 20 March 2014, *Y. Xiao & N. F. Fu LS-2841* (CSH!); • Yanling County, Taoyuandong Scenic Area, in valleys, humus soil, thickets, by the roadside, and in shaded places, 9 April 2014, *W. B. Liao et al. LXP13-5352* (SYS!); • Yanling County, Taoyuandong Scenic Area, on mountain slopes, in humus soil, by the roadside, and on rocks, 9 April 2014, *W. B. Liao et al. LXP13-5358* (SYS!); • Yanling County, Taoyuandong Scenic Area, on rocks, 9 April 2014, *W. B. Liao et al. LXP13-5370* (SYS!); • Yanling County, Taoyuandong Scenic Area, in shaded places, 9 April 2014, *W. B. Liao et al. LXP13-5375* (SYS!); • Zhangjiajie National Forest Park, Jinbian Stream, under the forest and by the ditch, elev. 670 m, 7 March 2015, *H. Zhou & J. L. Luo 15030704* (CSFI!); • Zhangjiajie National Forest Park, Jinbian Stream, by the ditch, elev. 680 m, 21 April 2015, *H. Zhou & J. L. Luo 15032516* (CSFI!); • Zhuzhou city, Yanling County, Taoyuandong Scenic Area, on sunny mountain slopes with humus soil, roadside, elev. 9 April 2014, *W. B. Liao et al. LXP13-5356* (SYS!). • Jiangxi: • Jinggangshan, Jinsinan, shaded understory by stream margin, elev. 450 m, 7 April 1983, *Department of Biology, Jiangxi University 83041* (PE!); • Lichuan County, Yanquan Forest Farm, Maixizhou, in the valley, elev. 600 m, 22 April 2015, *H. P. Tong 0002* (JJF!); • Lizhou, beneath rocks, elev. 900 m, 27 March 1965, *X. X. Yang 650088* (PE!) • Lushan, on Mountain slopes, elev. 600 m, 2 April 1978, *S. S. Lai et al. 0011* (LBG!); • Lushan, in the forest understory, elev. 1000 m, 6 August 1997, *Lai & Shan 1109* (NAS!); • Lushan: Hanpokou, Found in the forest understory, elev. 1000 m, 19 April 1992, *M. X. Nie 92040* (PE!); • Lushan, in the forest understory, elev. 1000 m, 24 April 1994, *M. X. Nie 92036* (PE!); • Wuning County, Luoxi, in the wetland beside the valley ditch, elev. 1050 m, 10 May 2011, *J. H. Zhang 1921* (JJF!); • Xiushui County, Heshi Town, at the edge of the valley forest, elev. 400 m, 28 April 1996, *L. X. Li 9604132* (JJF!); • Yifeng County, Lushan Mountain, Ma’er Peak, at the edge of the valley forest, elev. 600 m, 28 March 2009, *J. H. Zhang 1484* (JJF!); • Yifeng County, Lushan Nature Reserve, under the trees in the mountains, 26 April 1997, *S. S. Lai et al. 1917* (NAS!). • Shanxi: • Lingchuan County, Fenghuanggu, in canyon habitats and shady spots, elev. 912 m, 35°34'29.14"N, 113°22'40.0"E, 14 April 2014, *D. M. Kong k0078* (PE!); • Lingchuan County, Fenghuang Valley, in the shady place on the cliff of the canyon, elev. 912 m, 35°34'29"N, 113°22'46"E, 14 May 2014, *D. M. Zhang K0078* (SXU!); • Meixian, Tangyu Town, near Chuanxunliang, on rocky gravel areas, elev. 2500 m, 25 May 1971, *Z. B. Wang 19993* (WUK!); • Qinshui Country, Shunwangping, on the grassland of the mountaintop, elev. 2700 m, 2 May 2007, *J. Y. Yue 970* (HSIB!); • Qinyuan County, Lingbao mountain, on mountain slope, elev. 25 May 1959, *K. J. Guan & Y. L. Chen 327* (WUK!); • Qingshui,Within dense forests along mountain streams in valleys, elev. 1750 m, 12 June 1983, *Loess Archaeological Mission (Shanxi) 120* (WUK!); • Qinshui County, Zhongcun Town, Xiachuan Village, elev. 1750 m, 10 May 1981, *T. W. Liu & C. G. Li 491* (HSIB!); • Taiyuan City, Wanbailin District, on the way from Xiaoyaotou Village to Xingou Village, in the wetland by the roadside, 28 April 1959, *S. Y. Bao & S. J. Yan 229* (HSIB!); • Yicheng, Found in damp shrub understory, elev. 1800–2000 m, 8 May 1957, *Yellow River Survey Team 0024* (PE!); • Yuanqu County, Shunwangping, in the understory on mountain slopes, elev. 1300 m, 21 April 1982, *T. W. Liu & P. F. Zeng 106* (HSIB!); • Yuanqu County, Shunwangping, on mountain slope, 25 May 1959, *K. J. Guan & Y. L. Chen 327* (HSIB!); • Yuanqu County, Shunwangping, in the mountains, elev. 2153 m, 35°25'43.22"N, 111°57'30.17"E, 4 January 2014, *L. Wu et al. ZTS004* (BNU!); • Yuanqu County, Shunwangping, in the mountains, elev. 912 m, 35°25'43.22"N, 111°57'30.17"E, 4 January 2014, *L. Wu et al. ZTS005* (BNU!). • Shaanxi: • Mei County, northern foot of Taibai Mountain, in the grass on the mountain slope, elev. 359 m, 3 April 2006, *Z. J. Lin 06040305-2* (BNU!). • Sichuan: • *J. H. Xiong & Z. L. Zhou 90471* (IBSC!). • Zhejiang: • Hangzhou City, Lin’an District, Qingliangfeng National Nature Reserve, Shunxiwu, in the grass under the forest in the valley on the northwest slope, elev. 600 m, 14 April 1978, *Anonymous 405* (HTC!); • Hangzhou City, Lin’an District, Tianmushan, under rocks by paths in woods on southeast hillsides, 29 April 1957, *X. Y. He 21207* (PE!); • Hangzhou, Lin’an District, Tianmushan, on the mountain slope, elev. 1080 m, 1 May 1957, *X. Y. He 21375* (HHBG!); • Hangzhou, Lin’an District, Tianmushan, on mountain slopes and rocks, elev. 900 m, 25 April 1957, *X. Y. He 21056* (HHBG!); • Hsi-tienmu-shan, on forest edge, elev. 1080 m, 1 May 1957, *X. Y. He 21375* (NAS!); • Hsi-tienmu-shan, on forest edge, elev. 900 m, 26 April 1957, *X. Y. He 21056* (NAS!); • Hsi-tienmu-shan, 27 March 1928, *Anonymous 891* (PE!); • Hsi-tienmu-shan, 23 April 1936, *H. Migo s.n.* (PE!); • Hsi-tienmu-shan,1 April 1929, *Anonymous 37* (PE!); • Hsi-tienmu-shan, 30 April 1930, *Xihu Museum 43* (SHM!); • Hsi-tienmu-shan, 18 April 1929, *Anonymous D14* (PE!); • Quzhou City, Jiangshan City, Nianbadu Town, Xingdun Village, Tongwu Ancient Path, 18 April 2017, *X. Zhong et al. ZXX00308* (HIB!); • Quzhou City, Jiangshan City, Nianbadu Town, in subtropical evergreen and deciduous mixed forests, elev. 628.57 m, 28"N, 118°E, 13 March 2018, *B. J. Ge et al. GBJ05991* (CSH!); • Quzhou City, Jiangshan City, Nianbadu Town, in subtropical evergreen and deciduous mixed forests, elev. 671.88 m, 28"N, 118°E, 14 March 2018, *B. J. Ge et al. GBJ05872* (CSH!); • Xiwu, elev. 860 m, 9 May 1957, *X.Y. He 22673* (NAS!).

***Sinojohnstoniaruhuaii***: China. • Jiangxi: • Mt. Sanqing [San-qingshan], Jinshayulian waterfall, 28°55'N, 118°06'E, 560 m, 2 Apr. 2007, *Team of Mt. Sanqingshan of Sun Yat-sen University 19117* (Holotype, SYS); • Jiangxi: • Mt. Sanqing [San-qingshan], Jinshayulian waterfall, 28°55'N, 118°06'E, 560 m, 2 Apr. 2007, *Team of Mt. Sangingshan of Sun Yat-sen University 19108* (paratype, SYS); • Mt. Sanqing [San-qingshan], Jinshayulian waterfall, 560 m, 1 Apr. 2014, *Wen-bo Liao & Lei Wang 2014-L310, 2014-L314, 2014-L317* (Paratypes, SYS); • Mt. Sanqing [San-qingshan], Jinshayulian waterfall, 28°55.110'N, 118°05.835'E, 423 m, 2 Nov. 2013, *Wen-bo Liao & Lei Wang 2013-L048* (Paratype, SYS); • Mt. Sanqing [San-qingshan], Shiguling, 400 m, 3 Nov. 2013, *Wen-bo Liao & Lei Wang 2013-L007* (Paratype, SYS).

## Supplementary Material

XML Treatment for Sinojohnstonia henanensis

## ﻿Additional information

### Use of AI

No use of AI was reported.

### Author ORCIDs

Li-Qiong Jiang https://orcid.org/0000-0002-0781-8337

Yuan Luo https://orcid.org/0000-0001-6524-6317

Jian-Ping Gong https://orcid.org/0009-0000-3063-0925

Nai-Yu Zhang https://orcid.org/0009-0003-7563-3881

Pan Li https://orcid.org/0000-0002-9407-7740

Wen-Bo Liao https://orcid.org/0000-0001-6839-9511

Lu-Xian Liu https://orcid.org/0009-0003-8306-2257

### Data availability

All of the data that support the findings of this study are available in the main text.
